# Temporal Hierarchy and Context-Dependence of Quorum Sensing Signal in *Pseudomonas aeruginosa*

**DOI:** 10.3390/life12121953

**Published:** 2022-11-22

**Authors:** Stoyko Katzarov, Volker Behrends

**Affiliations:** The Centre for Integrated Research in Life and Health Sciences, University of Roehampton, London SW15 4JD, UK

**Keywords:** mass spectrometry, ESKAPE pathogens, *Pseudomonas aeruginosa*, quorum sensing, hierarchy, sub-MIC antibiotics

## Abstract

The Gram-negative bacterium *Pseudomonas aeruginosa* can cause infections in a broad range of hosts including plants, invertebrates and mammals and is an important source of nosocomial infections in humans. We were interested in how differences in the bacteria’s nutritional environment impact bacterial communication and virulence factor production. We grew *P. aeruginosa* in 96 different conditions in BIOLOG Gen III plates and assayed quorum sensing (QS) signaling over the course of growth. We also quantified pyocyanin and biofilm production and the impact of sub-inhibitory exposure to tobramycin. We found that while 3-oxo-C12 homoserine lactone remained the dominant QS signal to be produced, timing of PQS production differed between media types. Further, whether cells grew predominantly as biofilms or planktonic cells was highly context dependent. Our data suggest that understanding the impact of the nutritional environment on the bacterium can lead to valuable insights into the link between bacterial physiology and pathology.

## 1. Introduction

*Pseudomonas aeruginosa* is an important opportunistic pathogen that has been shown to cause infections in plants, invertebrates, and vertebrates [1]. In humans, the pathogen can cause eye, ear or toe infections in healthy, immunocompetent people. Importantly, in healthcare settings, *P. aeruginosa* is responsible for burn wound and catheter-associated urinary tract infections as well as ventilator- and general healthcare-associated pneumonia [2,3]. Further, *P. aeruginosa* has been associated with long, chronic infections of the airways of people with cystic fibrosis (CF) [4,5,6,7].

Part of its pathogenic success is due to *P. aeruginosa’s* metabolic flexibility [6,8], its high resistance to antibiotics [9] and its large array of virulence factors [6,10,11,12]. These factors include different types of protein secretion systems and secreted proteins [13]. Depending on the type of secretion system, proteins are either secreted into the cell’s environment or directly injected into the target cell. The complement of effector proteins, more specifically whether the cell carries ExoU (a phospholipase) or ExoS (a bi-functional toxin with GTPase-activating protein and adenosine diphosphate ribosyl transferase activity), determines whether strains of *P. aeruginosa* are invasive or cytotoxic [14,15,16]. Other virulence factors include siderophores (pyoverdine, pyochelin [17]), redox-active phenazines and their derivative pyocyanin [18] as well as the toxin cyanide [19].

The regulation of several of these virulence factors occurs via quorum sensing (QS) [20,21], particularly if the cells are in a planktonic state [6,12,21]. *P. aeruginosa* has three interconnected QS systems (the *lasR*, *rhlR* and the *pqs* systems), but several environmental and physiological cues are integrated into these systems [22]. The signaling molecules of the *las* and *rhl* systems are acyl-homoserine lactones (HSLs), namely 3-oxo-dodecanoyl-HSL (3oC12-HSL) and butyryl-HSL (C4-HSL), respectively. The *pqs* (or Pseudomonas quinolone signal) molecule is 2-heptyl-3-hydroxy-4-quinolone, but other, structurally related alkyl quinolone (AQ) compounds with specific biological functions exist [23,24]. QS controls up to 10% of gene expression of *P. aeruginosa* [25]. In planktonic cells, several virulence factors, e.g., elastase, pyocyanin and cyanide are QS-controlled. Conversely, the Type-3-secretion system is repressed by QS [26].

Several studies have investigated context-dependence of biofilm formation [27] and eradication [28], virulence factor production [8,18,29,30] and/or antimicrobial resistance [31,32,33,34], though usually in a limited number of conditions at one time-point. Here, we were interested to investigate the interaction of cell number/growth phase and growth environment on different QS systems to elucidate their impact on virulence factor and biofilm production. We performed intermittent sampling across the growth curves in 96 conditions in a BIOLOG GenIII plate and quantified the QS signal, the virulence factor pyocyanin (by mass spectrometry) and biofilm production after 24 h. We found that while QS hierarchy follows expected patterns in rich media, the expression of virulence factors and biofilm formation was highly context dependent.

## 2. Materials and Methods

*Pseudomonas aeruginosa* wild-type PA14, a strain originally isolated due to its ability to infect plants as well as vertebrates [1], was grown in Luria Bertani (LB) broth (10 g/L NaCl, 10 g/L tryptone and 5 g/L yeast extract) overnight at 37 °C, shaking at 150 rpm. From these, starter cultures were inoculated by 1:100 dilution and left to grow until they reached an optical density at 600 nm of 0.6. At that point, 2 mL of the culture was harvested by centrifugation (5 min, 3000× *g*, RT), the supernatant discarded and the pellet washed and resuspended in 1 mL 10 mM phosphate buffered saline, pH 7. The process was repeated, and the resuspended cells were mixed with 10 mL inoculation fluid A and inoculated into BIOLOG Gen III plates (both BIOLOG, Hayward, CA, USA).

At 3, 5.5, 8, 11, 14, 17 and 20 h, readings at OD_595_ (for metabolic activity/oxidative metabolism) and OD_750_ (cell number) were taken, and 25 µL of culture were sampled, centrifuged (2000× *g*, 2 min, RT) and stored at −20 °C for extraction of QS molecules. After 24 h, we also assayed surface-attached biofilm formation using the crystal violet method [35]. To assess the impact of sub-inhibitory exposure to tobramycin, treated plates were mixed with 0.8 µg/mL tobramycin at inoculation and sampled after 20 h only.

To extract QS compounds, 10 µL of the supernatants was mixed with 90 µL acetonitrile (LC-MS grade) containing 0.1% formic acid (*w*/*v*), 1 uM heptyl homoserine lactone and 4-cyclohexyl-2(1H)-quinolone (for normalization of HSLs and PQS, respectively; both Merck, Darmstadt, Germany) and frozen at −20 °C for 1 h. For analysis, 10 µL of each sample were transferred to a high-recovery plate and mixed with 90 µL LC-MS grade water (Fisher Scientific, Hampton, NH, USA).

Detection of QS compounds by tandem mass spectrometry was based on a method published by Ortori et al. [36]. Separation was achieved on a Waters Acquity H Class Ultra Performance Liquid Chromatography (UPLC) system using a Waters HSS T3 UPLC column (2.1 mm × 100 mm, 1.8 µm particle size, equipped with a HSS T3 VanGuard pre-column) maintained at 45 °C. The UPLC system used 0.1% formic acid (in water) with 0.1 mM EDTA as phase A and 0.1% formic acid (in acetonitrile) as phase B. The gradient was 1% B for 0.75 min, then up to 35% B at 1.5 min, 75% B at 3 min and 99% B at 3.75 min. At 4.25 min, the system switched back to 1% B, which was held for 0.75 min.

Mass spectra were obtained on a Waters TQSmicro triple quad mass spectrometer with electrospray ionization in positive mode using multiple reaction monitoring. The source potential was 3.5 kV with the source held at 450 °C and a desolvation gas flow of 650 L/h. Transitions and mass spectral parameters (cone and collision voltages) were originally taken from [36] and optimized by manual infusion of 10 µM standard (at 10 µL/min) for C4-HSL, 3-oxo-C12-HSL, pyocyanin, PQS and HHQ. Biological quality control samples were injected every eight samples to control for drift in instrument sensitivity. Data analysis, including baseline correction and QC batch correction was carried out in Matlab using in-house scripts (modified from [37]). 

## 3. Results

### General Growth Characteristics on BIOLOG Gen III

**Figure 1:**
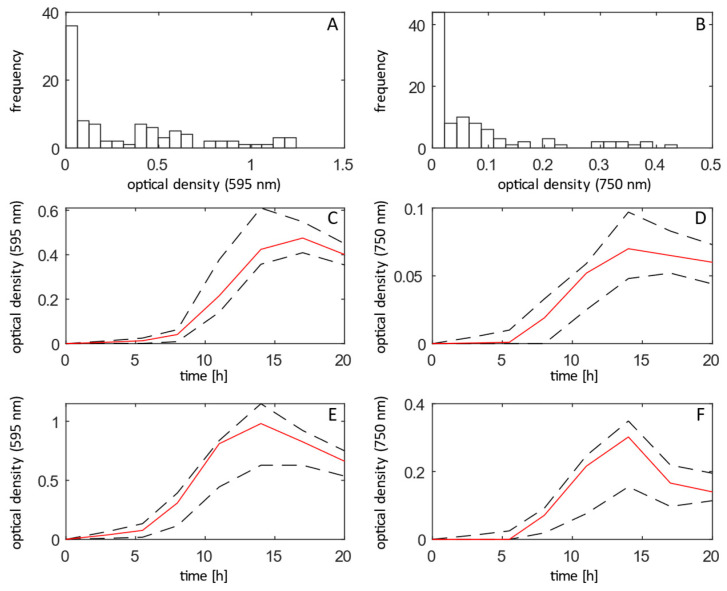
Optical density across growth conditions on a BIOLOG GenIII plate: (**A**) histogram of absorbance values recorded at 595 nm; (**B**) histogram of absorbance values recorded at 750 nm; (**C**) median (red line) and interquartile range (dashed black lines) of absorbance recorded at 595 nm for substrates supporting growth in wells A1–H9 (single carbon sources); (**D**) median (red line) and interquartile range (dashed black lines) of absorbance recorded at 750 nm for substrates supporting growth in wells A1–H9 (single carbon sources); (**E**) median (red line) and interquartile range (dashed black lines) of absorbance recorded at 595 nm for substrates supporting growth in wells A10–H12 (sensitivity assays); (**F**) median (red line) and interquartile range (dashed black lines) of absorbance recorded at 750 nm for substrates supporting growth in wells A10–H12 (single carbon sources).

BIOLOG Gen III plates contain 71 single carbon sources, one negative control (no carbon source), 23 sensitivity test conditions (rich media with a ‘stressor’) and one positive control (rich media only). While the single carbon source assays the ability of *P. aeruginosa* to grow on a substrate, the sensitivity test condition investigates the ability of the bacterium to overcome the ‘stressor’ (e.g., high salt stress or an antibiotic) in rich medium. To check which of these conditions supported oxidative metabolism (tetrazolium dye reduction) or measurable cell replication, we monitored optical densities at two wavelengths, 595 nm and 750 nm, respectively. Naturally, a different condition produced a high variety of outcomes with regards to growth rate and optical density reached after 20 h (Figure 1). Using exclusion criteria of reaching 10% of the recorded overall maximum value over the course of growth, we concluded that 52 conditions supported metabolic activity, 43 supported growth and 42 supported both, respectively (Figure 1, Table A1).

**Figure 2:**
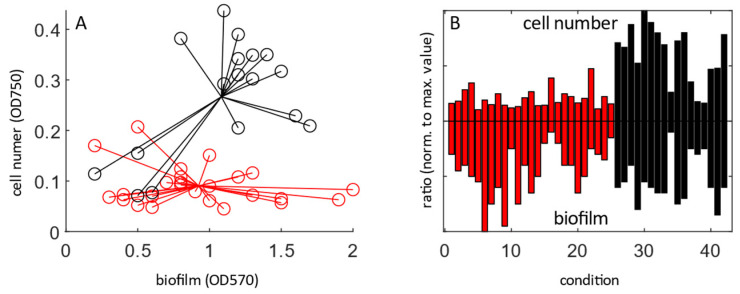
Context-dependent relationship between surface-attached biofilm and planktonic cell growth across conditions in the BIOLOG Gen III plate: (**A**) scatter graph of growth-supporting condition, red: single carbon sources, black: sensitivity assays; (**B**) bar chart displaying the ratio of planktonic and biofilm growth. Values were normalized to highest value across growth-supporting conditions, colors as (**A**).

Surface-attached biofilm was detectable in several conditions of the BIOLOG Gen III plate after 24 h of growth (Figure 2). There is no clear correlation between measured optical cell density (highest value at 750 nm) and biofilm development, but there is some grouping based on media type. Cultures grown on single carbon sources supported lower planktonic cell numbers than the sensitivity assays, which have a rich base medium (Figure 2A,B, Table A1). The median ratio of biofilm formation to planktonic growth was significantly higher in single carbon sources than in sensitivity assays (median 2.26 vs. 0.86 for maximum-normalised optical density values, respectively, *p* < 0.001, Student’s *t*-test). 

**Figure 3:**
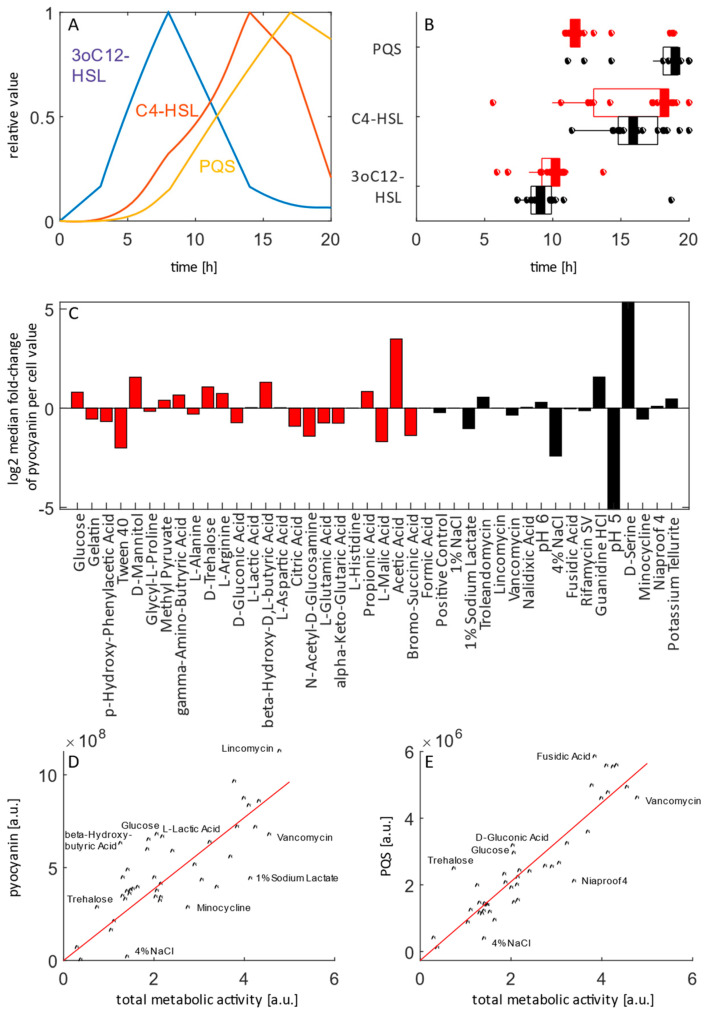
Temporal and cell-density dependence of QS and virulence factor production: (**A**) temporal hierarchy of QS system in rich media (positive control, well A10), normalized to highest value; (**B**) representation of temporal hierarchy of the three major QS systems across all growth-supporting conditions; red: single carbon sources; black: sensitivity assays; box and whisker plots represent median, interquartile range and extent of data range; (**C**) median fold change of pyocyanin-cell number ratio; single carbon sources and sensitivity assays were divided to their own median; red: single carbon sources; black: sensitivity assays; (**D**) scatter plot of total metabolic activity and pyocyanin produced over the course of growth; (**E**) scatter plot of total metabolic activity and PQS produced over the course of growth.

The temporal hierarchy of bacterial communication systems showed some variation between conditions (Figure 3A,B). In most wells, including the positive control (well A10), which contains a rich medium not unlike LB media routinely used for studies of pseudomonal QS, 3oC12-HSL was clearly produced before C4-HSL and PQS, in line with the established hierarchy of QS systems in P. aeruginosa [20]. The 3oC12-HSL was produced at fairly low cell densities and declined as peak cell numbers were reached, while C4-HSL and PQS levels increased more or less in line with cell number (median R2 to cell number (Pearson) 0.13, 0.86 and 0.83 for 3oC12-HSL, C4-HSL and PQS, respectively (Figure 1 and Figure 3)).

Interestingly, there is a clear difference with regards to stationary phase PQS levels. They increased with cell number in many conditions, but, in sensitivity assays, PQS levels rose with cell number and stayed near their maximum or even continued to increase for the recorded growth period. In most single carbon sources, PQS levels increased before cell number, peaked around 11 h post-inoculation and declined after that (Figure 3B). When investigating per-cell virulence factor production (pyocyanin per cell number) in the different condition sets, there are clear condition-dependent differences (Figure 3C). The per-cell pyocyanin production is about 20–30 fold higher in acetic acid-grown cultures or rich media supplemented with D-serine than in cultures grown at pH 5, respectively. When total levels of pyocyanin and PQS produced over the course of growth are compared and plotted against metabolic activity, it is evident that while overall correlation is good (R2 = 0.76 and 0.90, for pyocyanin and PQS, respectively), context-specific differences exist. For pyocyanin, production is generally lower than expected based on metabolic activity in a range of sensitivity assays (Figure 3D). For PQS, levels are higher than expected in wells with a sugar or disaccharide as the single carbon source (Figure 3D).

Finally, we were interested in the context-dependent impact of sub-inhibitory antibiotic exposure (Table 1). Metabolic activity did not exhibit a clear general trend (median log_2_ fold change across conditions of 0.02) but did exhibit a range of over six log_2_ units (log_2_ median fold change (log mfc) 3.99 for bromosuccinate to −2.16 for formic acid). Cellular growth and 3oC12-HSL exhibited similar profiles with median log mfc of 0.34 and 0.28, respectively, and ranges of log mfc from 3.76 (gluconic acid) to −1 (potassium tellurite) for cellular growth and log mfc from 4.33 (citric acid) to −1.98 (propionic acid) for 3oC12-HSL, respectively. 

However, for the two other QS systems as well as pyocyanin, there was a clear negative impact of tobramycin exposure. The impact was moderate for C4-HSL and PQS. The median across conditions were of −1.26 and −1.36 with ranges from −2.81 (propionic acid) to −0.07 (malic acid) and from −3.39 (potassium tellurite) to −0.13 (gelatin), respectively. In contrast, the impact on pyocyanin production was pronounced, with a median across conditions of −3.12 log_2_ units and ranges of log mfc from a value of −9.17 (propionic acid) to log mfc −1.1 (pH 5).

## 4. Discussion

We compared growth and quorum sensing signaling dynamics, virulence factor (pyocyanin) production and impact of sub-inhibitory antibiotic exposure across 96 different growth conditions in BIOLOG GenIII plates. We found several differences, but also similarities across growth-supporting conditions.

For most growth-supporting conditions, *P. aeruginosa* maintained the top-level QS hierarchy with 3oC12-HSL the first signal to be produced. The prominent exception was cells grown at pH 5, but this is likely connected to the very slow growth in the condition. A second similarity was that planktonic growth mostly depended on media type, not individual carbon source. Rich media-based sensitivity tests (wells A10-H12) in general supported growth to higher optical densities than single carbon sources within the time frame of the experiment. In contrast, surface-attached biofilm production over the first 24 h of growth did not correlate to media type.

Interestingly, there is a large variance in planktonic/biofilm partitioning in the different carbon source/conditions. The lowest relative levels of biofilm were found in cells grown in citric acid and rich media supplemented with 1% sodium lactate. This is in line with previous findings in the literature. *P. aeruginosa* cultures grown on citric acid have been shown to have altered biofilm morphology in a flow-cell model [38], and decreased biofilm production in a process depended on the TctD-TctE two component system [39]. Sodium lactate led to a decrease in biofilm production in *Shewanella putrefaciens* [40]. The process is dependent on LrbS-LrbA-LrbR, which is thought to have similar functions to RocS1, RocR and RocA1 in *P. aeruginosa* [41]. In *Pseudomonas*, RocS1A1R regulate the expression of Cup adhesion proteins, essential for biofilm formation [42]. 

The highest relative levels of biofilm were found in cultures grown on arginine and alanine, respectively. This agrees with previous literature, as arginine was found to be crucial for the formation of biofilms in bacterial–epithelial cell interaction models [43]. There are also mechanistic links that tie arginine to high biofilm production. It can be used as a nitrate donor [44], and high NO_3_ is linked to elevated biofilm production [45], while its denitrification product NO leads to biofilm dispersal [28]. Interestingly, the *rhl* QS system, which represses the transcription of denitrification genes [46] is partially regulated by arginine availability due to a rare arginine codon on the *rhlR* mRNA [47]. We did not see a change in biofilm levels (relative to the positive control) if the media was supplemented with D-Serine. D-amino acids have been suggested to lead to biofilm dispersal, though this has been disputed recently [48,49].

Temporal hierarchy for C4-HSL and PQS also differed due to media type, though with considerable spread. In minimal media, PQS levels peaked earlier than in rich media/sensitivity assays and decayed towards the end of the observed growth period. One explanation is the iron chelation ability of PQS [50]. The compound could be upregulated in minimal media and enzymatically degraded to increase iron availability in stationary phase [51]. An important caveat to consider is that we are effectively measuring a planktonic-biofilm hybrid and that the crystal violet-based method ignores floating cell aggregates, which might differ in their physiology from both planktonic cells and surface-attached biofilms [52]. Further, it has been recently suggested that during surface attachment, the regulatory cascade with 3oC12-HSL promoting C4-HSL and PQS production, does not fully apply [53,54,55,56,57]. Rather, the production of cytotoxic alkyl quinolones seems to be driven by a process involving type IV pili and the surface sensor PilY1 [56,58,59]. Finally, in established biofilms (the formation of which is also controlled by QS [20,21]), virulence is generally thought to be lower than in planktonic or surface-attaching states [9,60].

PQS has been suggested previously to have a multifaceted role in *P. aeruginsa*, acting—among others—as QS signal [23,24], an iron chelator [50], a live/death signal via oxidative stress in *Pseudomonas* itself [61], an initiator of oxidative stress in host cells [62], a photosensitizer [63] and/or a warning signal of antibiotic stress on population level [64]. In our setting, we compared oxidative metabolic activity to PQS levels and found that—while generally well correlated—higher than expected levels of PQS in cultures grown on sugars and lower than expected levels of PQS in some sensitivity conditions. Given the multitude of roles, changes in PQS are likely to seen due to different triggers and should be investigated further. 

Finally, we found that sub-inhibitory exposure to 1/10 × MIC tobramycin had marked impact on C4-HSL, PQS and pyocyanin production. Generally, virulence factor production is thought to be downregulated upon sub-inhibitory antibiotics exposure, often through downregulation of QS, though variety exists among studies [6,12]. Carbon sources were differentially impacted by antibiotic exposure, further confirming the impact of nutritional context on antibiotic tolerance [32,33,65].

## 5. Conclusions

Overall, our study highlights the context-dependence of bacterial QS, mode of growth and resistance. Understanding the specifics of these interactions can lead to improvements in bacterial treatment. Therefore, nutritional environments of infections sites should be surveyed and recreated in the laboratory to make models of infection more realistic. Future work should expand on this study using additional strains, e.g., clinical isolates from CF patients to take into account the evolutionary history in different niches.

## Figures and Tables

**Table 1:** Impact of sub-inhibitory exposure to tobramycin (0.8 µg/mL) on metabolic activity, cellular growth the three QS systems and pyocyanin production. Values are expressed as log_2_ fold change of the treated vs. the control samples.

		Activity	Cell Number	3oC12-HSL	C4-HSL	PQS	Pyocyanin
Glucose	C1	0.08	0.01	0.50	−0.73	−0.58	−2.69
Gelatin	E1	2.17	0.72	0.10	−0.55	−0.13	-1.67
p-Hydroxy-Phenylacetic Acid	G1	−1.27	1.08	−0.42	−1.43	−1.36	−3.74
Tween 40	H1	−0.78	0.03	0.61	−0.73	−0.83	−4.11
D-Mannitol	D2	0.13	−0.02	−1.01	−2.03	−1.83	−2.36
Glycyl-L-Proline	E2	0.33	1.53	0.14	−1.29	−0.79	−2.25
Methyl Pyruvate	G2	0.15	0.00	−0.14	−1.00	−0.63	−1.61
gamma-Amino-Butryric Acid	H2	−0.84	−0.27	0.42	−0.88	−1.22	−2.33
L-Alanine	E3	−0.08	0.82	1.62	−0.79	−1.26	−2.58
D-Trehalose	A4	−0.14	−0.02	−0.74	−1.74	−1.80	−2.97
L-Arginine	E4	0.13	1.88	0.88	−1.82	−1.97	−4.88
D-Gluconic Acid	F4	0.06	3.76	0.30	−1.55	−0.88	−3.27
L-Lactic Acid	G4	0.26	1.71	1.54	−1.01	−0.60	−3.44
beta-Hydroxy-D,L-Butyric Acid	H4	−0.71	0.70	0.86	−1.31	−1.46	−4.17
L-Aspartic Acid	E5	0.26	1.60	0.17	−0.81	−1.46	−2.16
Citric Acid	G5	−0.13	−0.32	4.33	−0.60	−1.29	−3.31
N-Acetyl-D-Glucosamine	B6	0.38	−0.03	0.56	−1.41	−1.00	−3.36
L-Glutamic Acid	E6	0.19	0.16	0.42	−2.14	−1.66	−4.23
alpha-Keto-Glutaric Acid	G6	−0.02	2.87	0.71	−1.73	−1.68	−4.03
L-Histidine	E7	0.34	1.12	−0.28	−1.67	−1.37	−2.75
Propionic Acid	H7	0.00	0.00	−1.98	−2.81	−2.27	−9.17
L-Malic Acid	G8	0.12	0.24	0.28	−0.07	−1.51	−4.70
Acetic Acid	H8	0.00	0.00	2.90	−1.42	−2.55	−7.23
Bromo-Succinic Acid	G9	3.99	0.00	0.21	−0.22	−0.38	−1.33
Formic Acid	H9	−2.16	0.00	−0.63	−1.44	−0.65	−4.73
Positive Control	A10	−0.19	0.18	0.34	−1.23	−1.71	−4.12
1% NaCl	B10	−0.17	0.15	−0.15	−0.13	−1.35	−2.17
1% Sodium Lactate	C10	0.13	0.43	0.66	−0.77	−1.27	−3.52
Troleandomycin	D10	0.42	1.64	−1.75	−1.40	−1.05	−1.81
Lincomycin	E10	0.00	0.00	0.55	−0.85	−1.30	−1.90
Vancomycin	F10	−0.04	0.04	0.29	−1.02	−1.48	−2.79
Nalidixic Acid	G10	0.15	0.56	0.50	−0.74	−1.36	−2.11
pH 6	A11	0.24	1.49	−0.56	−1.50	−1.53	−2.70
4% NaCl	B11	0.57	1.48	−0.28	−0.10	−0.56	−1.21
Fusidic Acid	C11	−0.10	0.44	−0.21	−1.71	−2.18	−4.03
Rifamycin SV	D11	0.03	0.58	−0.81	−1.92	−2.31	−3.92
Guanidine HCl	E11	0.32	2.58	−0.59	−0.90	−1.18	−2.27
pH 5	A12	−0.78	1.89	−0.88	−0.72	−0.88	−1.10
D-Serine	C12	−0.62	0.00	1.07	−0.51	−1.55	−2.67
Minocycline	D12	−0.20	0.48	1.38	−2.10	−1.75	−4.11
Niaproof 4	E12	−0.29	−0.11	−0.22	−1.29	−1.58	−3.88
Potassium Tellurite	G12	−0.41	−1.00	2.82	−1.66	−3.39	−5.51

## Appendix A

**Table A1:** An overview of the conditions of the BIOLOG Gen III plate and whether the met the criteria for supporting metabolic activity and/or cellular growth.

Name	Well	Metabolic Activity	Planktonic Growth	Both	Biofilm	Ratio bf/Cell
Negative Control	A1					
D-Raffinose	B1					
Glucose	C1	x	x	x	x	1.87
D-Sorbitol	D1					
Gelatin	E1	x	x	x	x	2.49
Pectin	F1				x	
p-Hydroxy-Phenylacetic Acid	G1	x	x	x	x	1.41
Tween 40	H1	x	x	x	x	1.45
Dextrin	A2					
Lactose	B2				x	
D-Mannose	C2				x	
D-Mannitol	D2	x	x	x	x	5.34
Glycyl-L-Proline	E2	x	x	x	x	5.27
D-Galacturonic Acid	F2	x				
Methyl Pyruvate	G2	x	x	x	x	5.04
gamma-Amino-Butyric Acid	H2	x	x	x	x	2.43
D-Maltose	A3					
D-Melibiose	B3					
D-Fructose	C3	x			x	
D-Arabino	D3	x			x	
L-Alanine	E3	x	x	x	x	6.59
L-Galactonic Acid Lactone	F3					
D-Lactic Acid Methyl Ester	G3					
alpha-Hydroxy-Butyric Acid	H3				x	
D-Trehalose	A4	x	x	x	x	2.10
alpha-Methyl-D-Glucoside	B4					
D-Galactose	C4					
myo-Inositol	D4					
L-Arginine	E4	x	x	x	x	5.75
D-Gluconic Acid	F4	x	x	x	x	1.90
L-Lactic Acid	G4	x	x	x	x	2.45
beta-Hydroxy-D,L-butyric Acid	H4	x	x	x	x	3.58
D-Cellobiose	A5					
D-Salicin	B5					
3-Methyl Glucose	C5					
Glycerol	D5	x			x	
L-Aspartic Acid	E5	x	x	x	x	1.39
D-Glucuronic Acid	F5	x				
Citric Acid	G5	x	x	x	x	0.26
alpha-Keto-Butyric Acid	H5				x	
Gentiobiose	A6					
N-Acetyl-D-Glucosamine	B6	x	x	x	x	1.20
D-Fucose	C6	x				
D-Glucose-6-PO4	D6					
L-Glutamic Acid	E6	x	x	x	x	1.62
Glucuronamide	F6	x				
alpha-Keto-Glutaric Acid	G6	x	x	x	x	1.80
Acetoacetic Acid	H6					
Sucrose	A7					
N-Acetyl-D-Mannosamine	B7					
L-Fucose	C7					
D-Fructose-6-PO4	D7	x			x	
L-Histidine	E7	x	x	x	x	3.95
Mucic Acid	F7					
D-Malic Acid	G7				x	
Propionic Acid	H7	x	x	x	x	2.43
D-Turanose	A8					
N-Acetyl-D-Galactosamine	B8					
L-Rhamnose	C8					
D-Aspartic Acid	D8					
L-Pyroglutamic Acid	E8	x			x	
Quinic Acid	F8				x	
L-Malic Acid	G8	x	x	x	x	0.53
Acetic Acid	H8	x	x	x	x	2.73
Stachyose	A9					
N-Acetyl Neuraminic Acid	B9					
Inosine	C9	x			x	
D-Serine_single	D9					
L-Serine	E9				x	
D-Saccharic Acid	F9					
Bromo-Succinic Acid	G9	x	x	x	x	1.58
Formic Acid	H9	x	x	x	x	0.96
Positive Control	A10	x	x	x	x	0.85
1% NaCl	B10	x	x	x	x	0.82
1% Sodium Lactate	C10	x	x	x	x	0.46
Troleandomycin	D10	x	x	x	x	1.53
Lincomycin	E10	x	x	x	x	0.55
Vancomycin	F10	x	x	x	x	0.67
Nalidixic Acid	G10	x	x	x	x	0.81
Aztreonam	H10					
pH 6	A11	x	x	x	x	0.94
4% NaCl	B11	x	x	x	x	0.38
Fusidic Acid	C11	x	x	x	x	1.03
Rifamycin SV	D11	x	x	x	x	0.87
Guanidine HCl	E11	x	x	x	x	0.70
Tetrazolium Violet	F11					
Lithium Chloride	G11					
Sodium Butyrate	H11				x	
pH 5	A12	x	x	x	x	1.70
8% NaCl	B12		x			
D-Serine	C12	x	x	x	x	1.54
Minocycline	D12	x	x	x	x	1.28
Niaproof 4	E12	x	x	x	x	1.78
Tetrazolium Blue	F12					
Potassium Tellurite	G12	x	x	x	x	0.77
Sodium Bromate	H12					
Total		52	43	42	57	
